# Why is the gender gap in life expectancy decreasing? The impact of age- and cause-specific mortality in Sweden 1997–2014

**DOI:** 10.1007/s00038-018-1097-3

**Published:** 2018-04-13

**Authors:** Louise Sundberg, Neda Agahi, Johan Fritzell, Stefan Fors

**Affiliations:** Aging Research Center, Karolinska Institute and Stockholm University, Gävlegatan 16, 13330 Stockholm, Sweden

**Keywords:** Life expectancy, Aging, Cause of death, Gender gap, Mortality

## Abstract

**Objectives:**

To enhance the understanding of the current increase in life expectancy and decreasing gender gap in life expectancy.

**Methods:**

We obtained data on underlying cause of death from the National Board of Health and Welfare in Sweden for 1997 and 2014 and used Arriaga’s method to decompose life expectancy by age group and 24 causes of death.

**Results:**

Decreased mortality from ischemic heart disease had the largest impact on the increased life expectancy of both men and women and on the decreased gender gap in life expectancy. Increased mortality from Alzheimer’s disease negatively influenced overall life expectancy, but because of higher female mortality, it also served to decrease the gender gap in life expectancy. The impact of other causes of death, particularly smoking-related causes, decreased in men but increased in women, also reducing the gap in life expectancy.

**Conclusions:**

This study shows that a focus on overall changes in life expectancies may hide important differences in age- and cause-specific mortality. It also emphasizes the importance of addressing modifiable lifestyle factors to reduce avoidable mortality.

## Introduction

The gender gap in life expectancy is a well-known and well-explored pattern. Women in Sweden have had, on average, a 3-year higher life expectancy at birth than men since the 1750s (HMD 2015). The female mortality advantages have, however, gone through different phases (Thorslund et al. 2013). There was a sharp rise in the gender gap during the 1950s, when mortality started to decline at a faster rate among Swedish women than among Swedish men. The gender gap was at its greatest in 1978, since then the gender gap in life expectancy has been continuously declining (HMD 2015). The reasons for this gender difference are thought to have both biological and non-biological origins. Biological differences in genetic structure and hormones are suggested to favor female life expectancy (Luy and Wegner-Siegmundt 2015; Oksuzyan et al. 2008). However, several studies have attributed the bulk (> 75%) of the gender gap in life expectancy to non-biological factors (e.g., behavior, life style, social roles) (Luy and Wegner-Siegmundt 2015; Oksuzyan et al. 2008), with higher mortality risk among men due to smoking, hazardous alcohol consumption, substance abuse and occupational risks (Loef and Walach 2012; Oksuzyan et al. 2008). A recent paper addressing the gender gap in life expectancy in Sweden also points to social change as a main driver of the historically observed changes in the gender gap (Hemström 2016). As for the currently ongoing decrease of the gender gap in life expectancy, there is some evidence suggesting that it could mostly be attributed to changing gender patterns of smoking (Pampel 2002, 2005); specifically, an increase in smoking among women and subsequent delayed health consequences (Lopez et al. 1994; Pampel 2006). The gender gap in Sweden is one of Europe’s lowest (Van Oyen et al. 2013). One way to enhance our understanding of the recent decrease in the gender gap in life expectancy is to decompose life expectancy by age- and cause-specific mortality. While previous studies have addressed gender-specific life expectancies in Sweden (Hemström 2016; Thorslund et al. 2013), they have not addressed the role of age- and cause-specific mortality. In this study, we analyze recent changes in life expectancy in Sweden by assessing a more comprehensive list of age- and cause-specific mortality in 1997 and 2014. We do this by analyzing the impact of (1) changes in age- and cause-specific mortality on the increase in life expectancy in men, (2) changes in age- and cause-specific mortality on the increase of life expectancy in women, and (3) changes in age- and cause-specific mortality on the gender gap in life expectancy.

## Methods

Information on underlying causes of death was collected from the Swedish National Cause of Death Register for 1997 and 2014 (National Board of Health and Welfare 2016). In this register, underlying cause of death is defined as “(a) the disease or injury which initiated the train of morbid events leading directly to death, or (b) the circumstances of the accident or violence which produced the fatal injury” (WHO 2004). All deaths are coded in accordance with the International Classification of Disease 10th version (ICD-10). The Swedish National Cause of Death Register covers the deaths of all people registered as living in Sweden, including deaths that occurred outside Sweden. It does not cover stillborn infants, people applying for asylum, people temporarily residing in Sweden, or emigrants who are no longer registered as living in Sweden (Statistic Sweden 2010). The amount of data not reported to the National Board of Health and Welfare and therefore missing from death certificates has increased over time, from 0.4% in 1997 to 1.0% in 2014 (National Board of Health and Welfare 2000, 2015). Similarly, the percentage of death certificates that lack a sufficiently specific cause of death has increased, from 1.8% in 1997 to 2.6% in 2014 (National Board of Health and Welfare 2015; Statistic Sweden 2010).

We restricted our analysis of underlying cause of death to eight main chapters and 15 sub-chapters of the ICD-10 (Table 1), which cover 91–92% of all deaths. These causes of death were chosen because of their impact on overall mortality and/or change in life expectancy. All other causes of death, 8–9%, were collapsed into one single group (“other”). We used 5-year age groups (e.g., 0–4, 5–9 years) with the exception of the oldest age group, which was “85 years or older”. We used these age groups because they are the groups for which official statistics are available.

**Table 1:** Age-adjusted mortality rates with 95% confidence interval for 23 causes of death in the population of Sweden in 1997 and 2014

Cause of death	Men		Women	
	1997 (95% CI)	2014 (95% CI)	1997 (95% CI)	2014 (95% CI)
I00–I99 Diseases of the circulatory system	709.3 (699.9–718.6)	401.5 (395.2–407.8)	449.8 (444.0–455.6)	283.2 (278.9–287.5)
I20–I25 Ischemic heart diseases	378.6 (371.9–385.3)	174.4 (170.3–178.5)	194.7 (190.9–198.6)	95.3 (92.8–97.7)
I60–I69 Cerebrovascular diseases	142.6 (138.4–146.8)	73.4 (70.7–76.1)	117.6 (114.6–120.6)	62.8 (60.8–64.8)
I30–I52 Other forms of heart disease	99.9 (96.3–103.5)	100.9 (97.6–104.1)	75.5 (73.1–77.)	78.5 (76.3–80.7)
C00–D48 Neoplasms	341.2 (334.9–347.4)	289 (283.9–294.1)	225.5 (221.2–229.8)	208.2 (204.3–212.1)
C15–C26 Malignant neoplasms of digestive organs	99.8 (96.5–103.2)	86 (83.2–88.7)	69.6 (67.2–72.0)	61.6 (59.5–63.7)
C61 Malignant neoplasm prostate	76.7 (73.6–79.7)	61.1 (58.6–63.5)	**	**
C50–C50 Malignant neoplasm of breast	0.3 (0.1–0.5)	0.1 (0.0–0.2)	32.5 (30.9–34.1)	26.5 (25.1–27.8)
C51–C58 Malignant neoplasms of female genital organs	**	**	27.5 (26.0–29.0)	22.6 (21.3–23.9)
C30–C39 Malignant neoplasms of respiratory and intrathoracic organs	54.6 (52.2–57.1)	43.1 (41.1–45.0)	26.6 (25.0–28.1)	36.1 (34.4–37.7)
C34 Malignant neoplasm of bronchus and lung	51.7 (49.3–54.0)	40.9 (39.0–42.8)	25.5 (24.0–27.0)	35.5 (33.9–37.1)
J00–J99 Diseases of the respiratory system	117.2 (113.3–121.1)	71.1 (68.4–73.7)	70.1 (67.8–72.5)	48.7 (46.8–50.5)
J09–J18 Influenza and pneumonia	62.2 (59.3–65.1)	25.6 (23.9–27.2)	41.8 (40.1–43.6)	14.2 (13.3–15.2)
J40–J47 Chronic lower respiratory diseases	42.5 (40.2–44.8)	31.9 (30.1–33.6)	21.2 (19.9–22.5)	28.5 (27.1–29.9)
V01–Y98 External causes of morbidity and mortality	74.7 (71.9–77.4)	69.4 (66.9–71.9)	33.2 (31.6–34.8)	33 (31.5–34.5)
X60–X84 Intentional self-harm	20.8 (19.4–22.2)	16.6 (15.4–17.8)	7.7 (6.9–8.5)	7.4 (6.6–8.5)
W00–W19 Falls	10.1 (9.0–11.1)	14.2 (13.0–15.4)	5.2 (4.5–5.8)	7.5 (6.8–8.2)
F00–F99 Mental and behavioral disorders	37.5 (35.4–39.7)	55.3 (52.9–57.7)	34 (32.4–35.6)	64.3 (62.3–66.3)
F03 Unspecified dementia	18 (16.4–19.6)	37.3 (35.3–39.3)	25.3 (23.9–26.6)	49.5 (47.8–51.3)
G00–G99 Diseases of the nervous system	22 (20.5–23.6)	41.9 (39.9–43.9)	19.9 (18.6–21.1)	41.8 (40.1–43.5)
G30 Alzheimer’s disease	6 (5.2–6.9)	20.1 (18.7–21.6)	7 (6.3–7.8)	26.2 (24.9–27.4)
A00–B99 Certain infectious and parasitic diseases	12.9 (11.7–14.2)	27.1 (25.5–28.8)	9 (8.1–9.8)	18.2 (17.1–19.3)
R00–R99 Symptoms, signs, abnormal clinical and laboratory findings, not elsewhere classified	26.7 (24.8–28.6)	30.1 (28.4–31.8)	25.9 (24.5–27.3)	29.3 (27.9–30.7)
Total	1451.1 (1438–1464)	1078.6 (1068–1089)	944.3 (936–953)	793.9 (787–801)

Causes of deaths are coded according to ICD-10 and are listed in a hierarchical order according to their impact on mortality

** Sex specific cancer for the other gender. Not applicable

### Analysis

Age-standardized mortality rates were obtained from the National Cause of Death Register. Confidence intervals were calculated with the method used by Chiang (1961). Number of deaths was obtained for both genders (male and female), both years (1997 and 2014) and for each age group (0–4, 5–9… 85 years or older). We used Arriaga’s method to decompose life expectancy by age and cause of death. In a first step, the difference in life expectancy was decomposed by the contribution from each age group. This was based on a direct, indirect and interaction effect. In a second step, the contributions from each separate age group were parted into the contributions from each specific cause of death. By summing the contributions from each cause of death across all age groups, the total contributions from any given cause were obtained (Arriaga 1984; Auger et al. 2014; Preston et al. 2001). We decomposed the difference in life expectancy between (1) men in 1997 and 2014, (2) women in 1997 and 2014, (3) men and women in 1997, and (4) men and women in 2014. This way, we estimated the impact of age- and cause-specific mortality on the (1) increase in male life expectancy; (2) increase in female life expectancy; (3) gender gap in life expectancy in 1997, and (4) gender gap in life expectancy in 2014. We based all life expectancy estimates on the remaining life expectancy for the 0–4 year age group.

## Results

Between 1997 and 2014, age-standardized all-cause mortality decreased by 26% in men and 16% in women (Table 1), and life expectancy increased from 73.1 years to 76.1 years in men (3.6 years) and 78.3–80.3 years in women (2 years). Hence, the gender gap in life expectancy decreased from 5.2 years in 1997 to 3.6 years in 2014. Mortality decline was found across all age groups (with some exceptions as a result of very low mortality rates with large fluctuations), but especially profound in those 60 years or older. Across all age groups, mortality declined more in men than in women, especially in those 60 years or older.

Table 2, column 1, shows the contribution of age-specific mortality to the change in male life expectancy between 1997 and 2014. Lower mortality in men 65 years or older accounted for 2.3 years of the increase. The results on cause-specific mortality (Table 3) show that reduced mortality from diseases of the circulatory system explained most of the increase in life expectancy and reduced mortality from ischemic heart disease explained the greatest fraction of the increase. Mortality rose from a few causes, which in turn had a negative impact on life expectancy.

**Table 2:** Age-specific contribution to the change in life expectancy between 1997 and 2014

Age	Male LE 1997–2014		Female LE 1997–2014		Sex gap LE 1997		Gender gap LE 2014		Gender gap change	
	Years	%	Years	%	Years	%	Years	%	Years	%
≥85	0.21	5.9	0.17	8.2	− 0.71	13.7	− 0.62	17.0	0.09	5.81
80–84	0.39	11.0	0.35	17.2	− 0.64	12.3	− 0.51	14.2	0.13	8.39
75–79	0.57	15.7	0.34	16.9	− 0.77	14.8	− 0.51	14.1	0.26	16.77
70–74	0.66	18.3	0.35	17.1	− 0.72	13.8	− 0.40	11.1	0.32	20.65
65–69	0.49	13.6	0.16	7.9	− 0.63	12.1	− 0.35	9.7	0.28	18.06
60–64	0.37	10.3	0.14	6.7	− 0.47	9.2	− 0.28	7.8	0.19	12.26
55–59	0.24	6.7	0.11	5.4	− 0.29	5.6	− 0.18	5.0	0.11	7.10
50–54	0.22	6.0	0.13	6.3	− 0.20	3.9	− 0.12	3.4	0.08	5.16
45–49	0.15	4.1	0.07	3.6	− 0.16	3.1	− 0.10	2.7	0.06	3.87
40–44	0.12	3.4	0.06	3.0	− 0.13	2.4	− 0.07	2.0	0.06	3.87
35–39	0.11	3.1	0.05	2.6	− 0.13	2.5	− 0.08	2.2	0.05	3.23
30–34	− 0.01	− 0.4	0.02	0.8	− 0.07	1.4	− 0.11	3.1	− 0.04	− 2.58
25–29	0.00	− 0.1	0.00	− 0.1	− 0.10	1.9	− 0.11	3.0	− 0.01	− 0.65
20–24	0.02	0.5	0.02	1.2	− 0.11	2.1	− 0.12	3.3	− 0.01	− 0.65
15–19	0.00	− 0.1	0.02	1.0	− 0.02	0.3	− 0.04	1.1	− 0.02	− 1.29
10–14	0.01	0.2	0.02	1.1	0.01	− 0.1	− 0.01	0.2	− 0.02	− 1.29
5–9	0.04	1.1	0.00	− 0.2	− 0.03	0.6	0.01	− 0.3	0.04	2.58
< 4	0.02	0.7	0.02	1.1	− 0.01	0.3	− 0.01	0.3	0.0	0.0
Total	3.59	100	2.04	100	− 5.17	100	− 3.62	100	1.55	100

The first column shows the increase in male life expectancy; the second, the increase in female life expectancy; the third, the gender gap in life expectancy in 1997; and the fourth, the gender gap in life expectancy in 2014. The negative values in columns three and four indicate male disadvantage. Sweden year 1997 and 2014

**Table 3:** The impact of cause-specific mortality on the change in life expectancy between 1997 and 2014

Cause of death	Male life expectancy 1997–2014		Female life expectancy 1997–2014		Gender gap life expectancy 1997		Gender gap life expectancy 2014		Change gender gap life expectancy 1997–2014	
	Years	%	Years	%	Years	%	Years	%	Years	%
I00–I99 Diseases of the circulatory system (excl. I20–I25, I60–I69, I30–I52)	0.28	7.8	0.19	9.4	− 0.25	4.8	− 0.09	2.6	− 0.16	10.2
I20–I25 Ischemic heart diseases	1.72	47.9	1.09	53.3	− 1.82	35.2	− 0.93	25.7	− 0.89	57.5
I60–I69 Cerebrovascular diseases	0.49	13.6	0.60	29.2	− 0.25	4.8	− 0.14	3.8	− 0.11	7.3
I30–I52 Other forms of heart disease	0.01	0.2	− 0.02	− 1.0	− 0.24	4.7	− 0.28	7.8	0.04	− 2.7
Total I00–I99 Diseases of the circulatory system	2.50	69.6	1.86	90.9	− 2.56	49.6	− 1.44	39.9	− 1.12	72.2
C00–D48 Neoplasms (excl. C15–C26, C61, C50, C51–C58, C30–C39, C34)	0.20	5.4	0.16	7.9	− 0.43	8.3	− 0.47	12.9	0.04	− 2.6
C15–C26 Malignant neoplasms of digestive organs	0.14	3.8	0.11	5.5	− 0.29	5.7	− 0.29	8.0	0.00	0.1
C61 Malignant neoplasm prostate	0.16	4.4	0.00	0.0	− 0.63	12.2	− 0.63	17.3	− 0.01	0.4
C51–C58 Malignant neoplasms of female genital organs	0.00	0.0	0.10	4.8	0.32	− 6.2	0.30	− 8.3	0.02	− 1.4
C50 Malignant neoplasm of breast	0.00	0.0	0.13	6.3	0.41	− 8.0	0.38	− 10.4	0.04	− 2.3
C30–C39 Malignant neoplasms of respiratory and intrathoracic organs (excl. C34)	0.01	0.3	0.01	0.3	− 0.02	0.4	− 0.02	0.5	0.00	0.2
C34 Malignant neoplasm of bronchus and lung	0.16	4.6	− 0.10	− 4.9	− 0.25	4.7	− 0.04	1.0	− 0.21	13.4
Total C00–D48 Neoplasms	0.67	18.6	0.40	19.8	− 0.88	17.1	− 0.76	21.1	− 0.12	7.9
J00–J99 Diseases of the respiratory system (excl. J09–J18, J40–J47)	0.00	− 0.1	0.01	0.4	− 0.05	1.0	− 0.08	2.3	0.03	− 2.0
J09–J18 Influenza and pneumonia	0.18	5.0	0.26	12.9	− 0.15	2.9	− 0.12	3.3	− 0.03	2.2
J40–J47 Chronic lower respiratory diseases	0.09	2.5	− 0.06	− 2.8	− 0.15	2.9	− 0.02	0.6	− 0.13	8.3
Total J00–J99 Diseases of the respiratory system	0.27	7.4	0.21	10.5	− 0.36	6.9	− 0.22	6.2	− 0.13	8.5
V01–Y98 External causes of morbidity and mortality (excl. X60–X84, W00–W19)	0.07	1.8	0.04	2.0	− 0.46	8.9	− 0.47	13.1	0.01	− 0.9
X60–X84 Intentional self-harm	0.10	2.8	0.01	0.7	− 0.26	5.0	− 0.21	5.8	− 0.05	3.1
W00–W19 Falls	0.00	− 0.1	− 0.02	− 0.8	− 0.06	1.2	− 0.08	2.3	0.02	− 1.4
Total V01–Y98 External causes of morbidity and mortality	0.16	4.6	0.04	2.0	− 0.78	15.1	− 0.77	21.2	− 0.01	0.8
F00–F99 Mental and behavioral disorders (excl. F03)	0.16	4.4	− 0.03	− 1.4	− 0.18	3.5	− 0.05	1.3	− 0.13	8.6
F03 Unspecified dementia	− 0.08	− 2.3	− 0.22	− 10.8	0.05	− 0.9	0.11	− 3.1	− 0.07	4.3
Total F00–F99 Mental and behavioral disorders	0.08	2.1	− 0.25	− 12.2	− 0.14	2.6	0.07	− 1.8	− 0.20	13.0
G00–G99 Diseases of the nervous system (excl. G30)	− 0.02	− 0.5	− 0.02	− 0.9	− 0.04	0.8	− 0.07	2.0	0.03	− 1.9
G30 Alzheimer’s disease	− 0.06	− 1.8	− 0.18	− 8.7	0.01	− 0.2	0.06	− 1.7	− 0.05	3.2
Total G00–G99 Diseases of the nervous system	− 0.08	− 2.3	− 0.20	− 9.6	− 0.03	0.6	− 0.01	0.3	− 0.02	1.3
A00–B99 Certain infectious and parasitic diseases	− 0.06	− 1.7	− 0.09	− 4.2	− 0.05	0.9	− 0.10	2.7	0.05	− 3.3
R00–R99 Symptoms, signs, abnormal clinical and laboratory findings, not elsewhere classified	− 0.07	− 2.1	− 0.05	− 2.7	− 0.03	0.7	− 0.06	1.8	0.03	− 1.9
Other	0.14	3.9	0.11	5.4	− 0.34	6.6	− 0.32	8.8	− 0.02	1.4
Total	3.59	100	2.04	100	− 5.17	100	− 3.62	100	− 1.55	100

The first column shows the increase in male life expectancy; the second, the increase in female life expectancy; the third, the sex gap in life expectancy in 1997; the fourth, the sex gap in life expectancy in 2014; and the fifth, the change in sex gap between 1997 and 2014. Sweden year 1997 and 2014

Causes of deaths are coded according to ICD-10. Negative values in the first two columns indicate a negative impact on life expectancy. Negative values in the third and fourth columns indicate male disadvantage. Negative values in the fifth column indicate a reduction in the gender gap, and positive values indicate an increase in the gender gap. Causes of deaths listed in a hierarchical order according to their impact on mortality

Table 2, column 2, shows that reduced mortality in women 65 years or older accounted for 1.37 years of the total increase in female life expectancy. The results on cause-specific mortality (Table 3) show that reduced mortality from diseases of the circulatory system explained most of the increase in life expectancy, and reduced mortality from ischemic heart disease explained the greatest fraction of the increase. The increased mortality from certain causes observed in men was also observed in women. However, women also experienced an increase in smoking-related mortality (malignant neoplasms of respiratory and intrathoracic organs, malignant neoplasm of bronchus and lung, and chronic lower respiratory diseases), mortality from falls, and mortality from mental and behavioral disorders. Smoking-related mortality increased mainly in those 65 years or older; mortality from the other causes increased mainly in those 60 years or older. In total, women 80 years or older accounted for the bulk (82%) of the increased mortality from these causes (results not shown).

### Gender gap in life expectancy

**Fig. 1:**
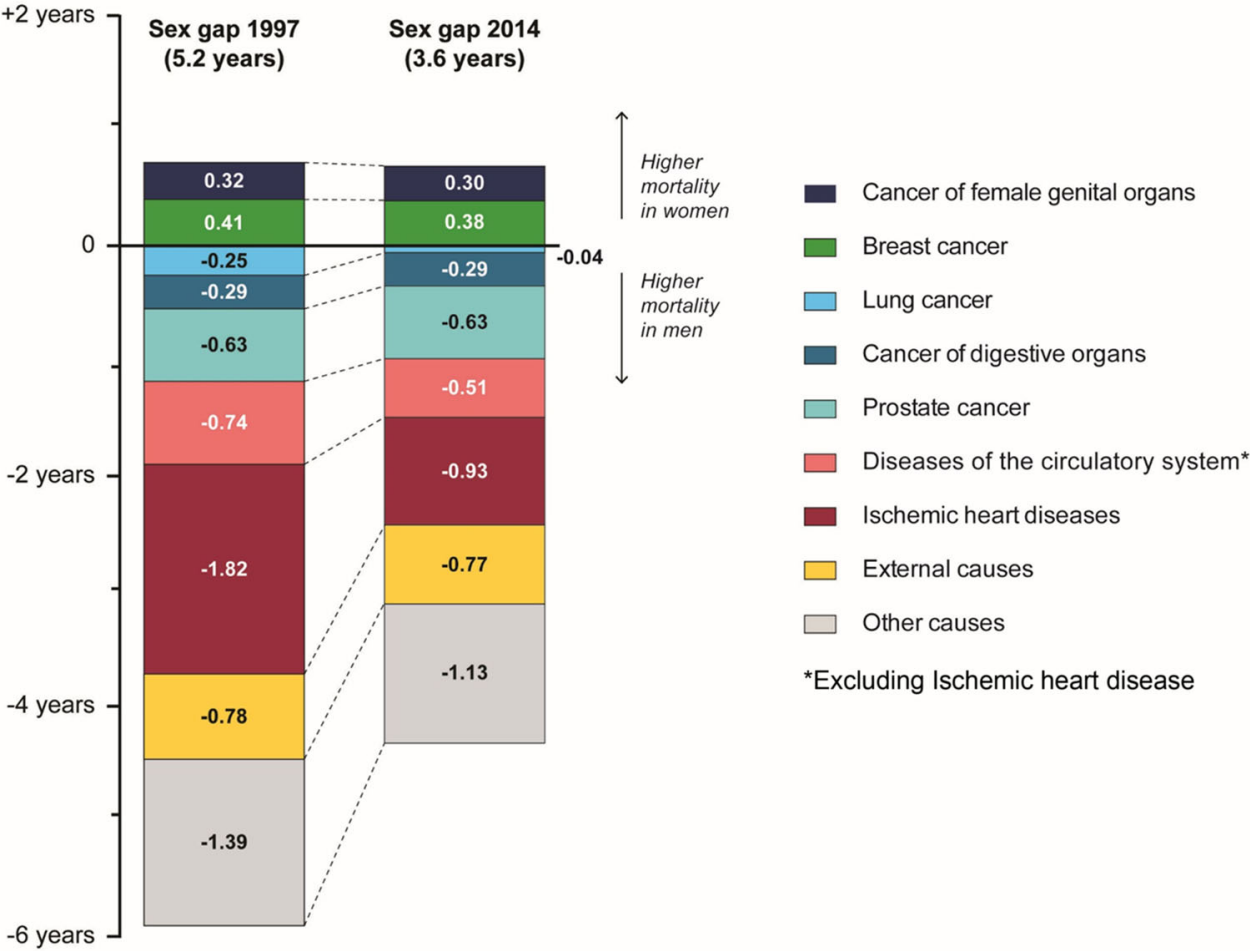
The gender gap in life expectancy in 1997 and 2014 decomposed by the eight main causes of death and other causes of death. Values above zero indicate higher mortality in women, and values below zero indicate higher mortality in men. The sum of the negative and positive values results in a gender gap of 5.2 years in 1997 and 3.6 years in 2014

Between 1997 and 2014, the gender gap in life expectancy decreased by 1.55 years. Figure 1 shows the main causes of death that explained most of the gender gap in each year. The decreasing gap resulted from three patterns. First, male mortality decreased at a faster rate than female mortality. Second, male mortality from certain causes of death decreased, whereas female mortality from those causes increased. Third, mortality from a few specific causes increased in both genders, but increased more in women. The first pattern (male mortality decreased at a faster rate than female mortality) was the main driver of the decrease in the gender gap. Essentially all main ICD-10 chapters of underlying cause of death show this trend. As seen in Fig. 1, reduced mortality from diseases of the circulatory system, especially from ischemic heart disease, had the largest impact on the reduction in the gender gap. However, when we decomposed the result by ICD-10 sub-chapters, more details emerged. Smoking-related deaths (from lung cancer and chronic lower respiratory disease), deaths from falls, and deaths from mental and behavioral disorders decreased in men but increased in women. Mortality from unspecified dementia and Alzheimer’s disease increased in both genders, but had a larger negative impact on female life expectancy than male life expectancy.

Analysis of the impact of age-specific mortality on the gender gap in 1997 showed that mortality in people 65 years or older had the greatest impact on the overall gender gap. By 2014, a clear shift had occurred. Mortality in people 85 years or older had the greatest impact on the overall gender gap, although the gender gap also diminished in this age group.

## Discussion

In this study, we assessed the impact of age- and cause-specific mortality on the increasing life expectancy among men and women, and on the decreasing gender gap in life expectancy, between years 1997 and 2014. We found that lower mortality in men 60–84 years explained most of the increase in men’s life expectancy, and that lower mortality in women 70–84 years explained most of the increase in women’s life expectancy. The decreasing gender gap was primarily the result of a more rapid mortality decline from circulatory diseases among men than women, especially in those 70–74 years.

These results should be viewed in light of a number of limitations. First, everyone 85 years or older were collapsed in a single age group, since the official data used set this boundary for the oldest age group, and a large proportion of all deaths happened in this group. During the study period, the number and proportion of deaths occurring in this age group increased, as did the mean age of death (HMD 2015). Hence, the description of the mortality pattern among the oldest old is less detailed than the description of the mortality patterns in younger age groups. The second limitation regards the classification of the underlying cause of death. The number of missing death certificates and the number of death certificates with insufficient information have increased over time. However, missing data are still rare (0.4–2.6%). Third, previous studies have found major coding errors in the main ICD chapters of death certificates (Alfsen and Maehlen 2012; Mieno et al. 2016). Such coding errors are especially relevant in studies of older people, because multimorbidity, which is common in the older population, increases the uncertainty of death certificates (Lahti and Penttilä 2003; Mieno et al. 2016). Although misclassification of the underlying cause of death is a substantial problem at the individual level, such misclassification is less of a problem at the aggregated level, because the errors tend to balance each other (Statistic Sweden 2010). Sweden’s decreased autopsy rate could also negatively impact the accuracy of the diagnoses of underlying causes of death (Alfsen and Maehlen 2012; Lindström et al. 1997). The autopsy rate declined during the study period and is generally lower in the older population (National Board of Health and Welfare 2000, 2015). However, declining autopsy rates do not necessarily imply increasing errors, as diagnostics and health records have improved in parallel (Statistic Sweden 2010).

We chose the study period specifically to facilitate comparisons of the causes of death. Sweden shifted from the ICD-9 to the ICD-10 in 1997, so by selecting 1997 as the initial study year, we minimized potential classification errors that could have arisen from a shift in coding the underlying cause of death. Another strength of the study is that we focused on main chapters from the ICD-10 and included a limited number of sub-chapters. Because coding errors increase with increasing level of diagnostic detail (Lahti and Penttilä 2001; Statistic Sweden 2010), this choice reduced the risk for such errors. However, we still assessed a more comprehensive list of causes of death than previous studies, which have either focused on a specific age range (Glei and Horiuchi 2007; Vollset 2013), fewer causes of death (Klenk et al. 2016; Trovato and Lalu 1998), or avoidable mortality (Westerling 1992, 2003; Westerling et al. 1996).

With regard to age-specific mortality, a previous study that addressed cohort differences in mortality found that in cohorts born in and after 1880, the gender gap in life expectancy was mainly the result of higher mortality among men aged 50–70 years (Beltrán-Sánchez et al. 2015), and other period-specific studies have found the same (Glei and Horiuchi 2007; Klenk et al. 2016; Trovato and Lalu 1998). In this study, we found a somewhat different pattern. In 1997, the main explanation for the gender gap in mortality was the higher mortality rate in men 65 years or older. In 2014, mortality in the age groups 65 and above was still important, but the main contribution came from those 85 years or older. Given the ongoing increase in life expectancy in old age, the growing impact of mortality at older ages on the gender gap is not surprising.

Our results on cause-specific mortality are in line with what others have found; namely, that most of the increased life expectancy and most of the decreased gender gap in life expectancy is the result of reduced mortality from circulatory diseases. Our results show that decreased mortality from circulatory diseases accounted for 70% of the reduced gender gap, and that ischemic heart disease explained the vast majority of this decrease. We know that lifestyle has a profound impact on the occurrence of circulatory disease and of cardiovascular disease (Kones and Rumana 2014), and that smoking is a major contributor (Lim et al. 2012). Given that there is a gender difference in lifestyle behaviors, especially in smoking (Peters et al. 2014), but also in diet, overweight and obesity (Institute for Health Metrics and Evaluation (IHME) 2016), the reduced gender gap in life expectancy could be a reflection of diminishing gender differences in lifestyles. However, while decreased smoking has had a positive impact on life expectancy through the reduction of diseases in the circulatory system, the increasing prevalence of obesity could have a counteracting effect. Obesity has increased in both men and women in Sweden (Juul and Hemmingsson 2010), and it is a contributing factor to many causes of death (Barbieri et al. 2017). Hence, although not stated as the underlying cause of death, the increasing prevalence of obesity has probably had a negative impact on life expectancy development among both men and women. To what degree it has had an impact on the gender gap, and in what direction, we do not know as of yet, but the prevalence of obesity has been found to be similar across the genders (Juul and Hemmingsson 2010). Although smoking was initially much more prevalent among men, the prevalence of smoking has become higher among women than men since the 1990s (Midlöv et al. 2014; Patja et al. 2009). Our results show that mortality from lung cancer and lower respiratory disease decreased in men but increased in women [about 90% of all deaths from chronic lower respiratory disease in Sweden in 2014 were due to chronic obstructive pulmonary disease (National Board of Health and Welfare 2016)]. Thus, the impact of lung cancer and of chronic lower respiratory disease on the gender gap in life expectancy decreased overall, and was attributable to both decreased mortality in men and increased mortality in women.

The results also showed a rise in mortality from certain causes of death, which diminished the increases in life expectancy by a total of − 0.3 years in men and − 0.8 years in women. This was especially evident for unspecified dementia, Alzheimer’s disease and mortality from falls. Yet, some of the observed increases in cause-specific mortality should be interpreted with caution. Most of the observed mortality increases from these causes of death occurred in the oldest age groups. On the one hand, there are more inaccuracies on the death certificates of the oldest age groups (Lahti and Penttilä 2003), and one known problem is an underreporting of Alzheimer’s disease as the cause of death (Ganguli and Rodriguez 1999; Garcia‐Ptacek et al. 2016). This could mean that the current analyses underestimated the increase in Alzheimer’s disease as a cause of death among the oldest old, both in 1997 and in 2014. On the other hand, improved diagnostic tools and increased awareness might have reduced the problem of underreporting of certain diseases, as has happened with falls (Kharrazi et al. 2015). Thus, at least part of the increase in falls and in Alzheimer’s disease as causes of death might be due to more accurate reporting in 2014 than 1997. We have no reason to suspect that the reporting procedure would affect men and women differently, and therefore we do not believe that this has had a substantial impact on the gender gap. Moreover, the increase of deaths from certain causes is likely to reflect a real change, rather than just changes in the coding procedure. When some causes of death decrease, others tend to increase, as competing risks. Increased survival, especially from diseases of the circulatory system, has allowed an increasing proportion of the population to reach very old age, and thereby to be at risk for Alzheimer’s disease and other age-related diseases. Similarly, cancer mortality has decreased the last decades, and it also declines in very old age (Liu and Liu 2016), thus opening up for the increase of other causes of death.

Researchers have consistently pointed towards the different evolution of smoking behavior among men and women as an important explanation for the decreasing gender gap in life expectancy (Hemström 2016; Luy and Wegner-Siegmundt 2015; Pampel 2002, 2005). However, it seems like the impact of smoking only plays a minor role for the current gender gap. Whereas it accounted for about 20% of the gap from the 1950s through the 1980s, it only accounted for about 5% of the gap in 2013 (Luy and Wegner-Siegmundt 2015; Valkonen and Van Poppel 1997). Our study did not specifically address smoking-related mortality, but its diminishing impact on the remaining gender gap is evident in the finding that mortality from chronic lower respiratory disease and lung cancer decreased in men but increased in women. Although mortality from smoking contributes to reducing the gender gap, smoking still remains an important public health target since it has a strong negative impact on health and mortality, and especially since mortality from smoking has increased among women. Yet, our results put forward the importance of both biological factors and lifestyle factors other than smoking for the explanations of the gender gap in life expectancy. Previous studies of the gender gap in life expectancy in Sweden have emphasized the importance of social conditions and social change (Hemström 2016; Thorslund et al. 2013). Our findings underscore this emphasis. Thus we would like to highlight the fact that other lifestyle factors besides smoking may need to be targeted in order to maintain a positive development of life expectancy for both genders, and a continuously decreasing gender gap. Hence, future studies should put emphasis on the impact of a broader range of modifiable life style factors and avoidable mortality in order to understand the ongoing trend in increasing life expectancy, the decreasing gender gap in life expectancy, as well as the scope for future improvements.
